# Clinical characteristics, treatment, and outcomes in CIED-related vs. left-sided infective endocarditis: a nationwide study from the NIDUS registry

**DOI:** 10.1093/europace/euag075

**Published:** 2026-04-09

**Authors:** Amna Alhakak, Peter Laursen Graversen, Katra Hadji-Turdeghal, Jacob Eifer Møller, Niels Eske Bruun, Anne-Christine Ruwald, Peter Godsk Jørgensen, Andreas Dalsgaard Jensen, Jeppe K Petersen, Mohammed Bakir Ahmad Lafta, Kasper Høtoft Bengtsen, Melanie Vuong Le, Kasper Karmark Iversen, Jonas Agerlund Povlsen, Claus Ernst Moser, Morten Holdgaard Smerup, Jannik Helweg-Larsen, Henning Bundgaard, Lauge Østergaard, Lars Køber, Emil Loldrup Fosbøl

**Affiliations:** Department of Cardiology, Copenhagen University Hospital—Rigshospitalet, Blegdamsvej 9, 2100 Copenhagen, Denmark; Department of Cardiology, Copenhagen University Hospital—Rigshospitalet, Blegdamsvej 9, 2100 Copenhagen, Denmark; Department of Cardiology, Copenhagen University Hospital—Rigshospitalet, Blegdamsvej 9, 2100 Copenhagen, Denmark; Department of Cardiology, Copenhagen University Hospital—Rigshospitalet, Blegdamsvej 9, 2100 Copenhagen, Denmark; Research Unit of Cardiology, Department of Cardiology, Odense University Hospital, Odense, Denmark; Faculty of Health Sciences, University of Southern Denmark, Odense, Denmark; Department of Cardiology, Zealand University Hospital, Roskilde, Denmark; Department of Clinical Medicine, University of Copenhagen, Copenhagen, Denmark; Department of Clinical Medicine, Aalborg University, Aalborg, Denmark; Department of Cardiology, Copenhagen University Hospital—Rigshospitalet, Blegdamsvej 9, 2100 Copenhagen, Denmark; Department of Cardiology, Copenhagen University Hospital—Herlev and Gentofte, Herlev, Denmark; Department of Cardiology, Copenhagen University Hospital—Rigshospitalet, Blegdamsvej 9, 2100 Copenhagen, Denmark; Department of Cardiology, Copenhagen University Hospital—Rigshospitalet, Blegdamsvej 9, 2100 Copenhagen, Denmark; Department of Cardiology, Copenhagen University Hospital—Rigshospitalet, Blegdamsvej 9, 2100 Copenhagen, Denmark; Department of Cardiology, Zealand University Hospital, Roskilde, Denmark; Department of Cardiology, Zealand University Hospital, Roskilde, Denmark; Department of Clinical Medicine, University of Copenhagen, Copenhagen, Denmark; Department of Emergency Medicine, Copenhagen University Hospital—Herlev and Gentofte, Herlev, Denmark; Department of Cardiology, Aarhus University Hospital, Aarhus N, Denmark; Department of Clinical Microbiology, Copenhagen University Hospital—Rigshospitalet, Copenhagen, Denmark; Department of Immunology and Microbiology, University of Copenhagen, Copenhagen, Denmark; Department of Cardiothoracic Surgery, Copenhagen University Hospital—Rigshospitalet, Copenhagen, Denmark; Department of Infectious Diseases, Copenhagen University Hospital—Rigshospitalet, Copenhagen, Denmark; Department of Cardiology, Copenhagen University Hospital—Rigshospitalet, Blegdamsvej 9, 2100 Copenhagen, Denmark; Department of Clinical Medicine, University of Copenhagen, Copenhagen, Denmark; Department of Cardiology, Copenhagen University Hospital—Rigshospitalet, Blegdamsvej 9, 2100 Copenhagen, Denmark; Department of Cardiology, Copenhagen University Hospital—Rigshospitalet, Blegdamsvej 9, 2100 Copenhagen, Denmark; Department of Clinical Medicine, University of Copenhagen, Copenhagen, Denmark; Department of Cardiology, Copenhagen University Hospital—Rigshospitalet, Blegdamsvej 9, 2100 Copenhagen, Denmark; Department of Clinical Medicine, University of Copenhagen, Copenhagen, Denmark

**Keywords:** Infective endocarditis, Valvular heart disease, Implantable cardioverter–defibrillator, Pacemaker, Epidemiology

## Abstract

**Aims:**

Cardiac implantable electronic device (CIED)-related infective endocarditis (IE) presents distinct diagnostic and therapeutic challenges due to its unique characteristics and limited evidence supporting guidelines compared with left-sided valvular IE. We aimed to examine clinical characteristics, treatment, and mortality in patients with CIED-related IE vs. left-sided valvular IE.

**Methods and results:**

We included patients with first-time IE using nationwide data from the NatIonal Danish endocarditis StUdieS (NIDUS) registry (2016–2021) and categorized them into isolated CIED-related IE without concomitant valvular IE and left-sided valvular IE. A total of 340 patients with isolated CIED-related IE and 2510 patients with left-sided IE were included. Patients with CIED-related IE vs. left-sided IE were older (76.1 vs. 73.2 years), and a higher proportion were males (78.5% vs. 65.9%), had diabetes (32.9% vs. 21.9%), heart failure (46.2% vs. 11.7%), *Staphylococcus aureus* (37.1% vs. 30.7%), coagulase-negative staphylococci (11.2% vs. 6.4%), and culture-negative IE (12.1% vs. 7.8%). However, fever at admission was lower in CIED-related IE (55.4% vs. 61.5%). Cardiac implantable electronic device removal was performed in 78.2% of patients with CIED-related IE. The 6-month cumulative incidence of mortality was 20.4% (95% CI: 16.2–24.9%) in CIED-related IE and 26.8% (95% CI: 25.1–28.6%) in left-sided IE (*P* = 0.009). In a multivariable Cox regression model, CIED-related IE was associated with lower 6-month mortality compared with left-sided IE (adjusted HR: 0.52 [95% CI: 0.40–0.68], *P* < 0.001).

**Conclusion:**

In this nationwide study, patients with CIED-related IE were distinctly different from those with left-sided IE. Staphylococci were more prevalent, and despite higher age and differences in comorbidities, mortality was lower.

## Graphical Abstract

**Graphical Abstract:**
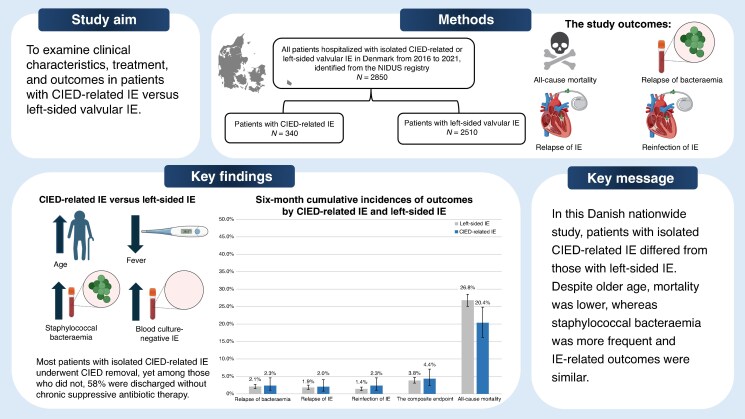
Overview of the study design, patient population, and main findings in patients with CIED-related IE compared with left-sided IE. CIED, cardiac implantable electronic device; IE, infective endocarditis. Created with BioRender.com.

What’s new?

- In this nationwide Danish cohort, patients with isolated CIED-related IE were older, had a distinct comorbidity profile, and were less likely to present with fever than patients with left-sided IE.
- Staphylococcal bacteraemia and culture-negative IE were more prevalent in isolated CIED-related IE than in left-sided IE.
- Six-month cumulative incidences of relapse of bacteraemia, relapse of IE, and reinfection of IE were similar between groups. However, despite older age, patients with isolated CIED-related IE had lower all-cause mortality, which remained substantial at approximately 20%.
- While most patients with CIED-related IE underwent CIED removal, more than half of those who did not undergo removal were discharged without chronic suppressive antibiotic therapy.
- These findings support isolated CIED-related IE as a distinct clinical entity and may inform more focused diagnostic and preventive strategies.

## Introduction

Isolated cardiac implantable electronic device (CIED)-related infective endocarditis (IE) is associated with significant morbidity and mortality.^1–3^ However, clinicians are faced with diagnostic and therapeutic challenges due to limited scientific evidence within this field compared with left-sided valvular IE.^4,5^

Cardiac implantable electronic device-related IE is often underdiagnosed or diagnosed late in clinical practice owing to its non-specific clinical presentation and the limitations of current diagnostic criteria and tools.^5^ In clinical practice, management of CIED-related IE is mainly guided by approaches developed for left-sided IE.^4,5^ However, direct comparative data between these conditions remain scarce and are limited to a single-centre study of 274 patients, which reported higher in-hospital mortality in left-sided IE (35%) than in CIED-related IE (28%), without a statistically significant difference.^6^

Despite similarities in clinical presentation, important distinctions between CIED-related and left-sided IE are not fully addressed in the existing literature and may affect timely and accurate diagnosis, antimicrobial treatment, and clinical decision-making. In this Danish nationwide study, we aimed to examine clinical and microbiological characteristics, treatment strategies, IE-related outcomes, and all-cause mortality in an unselected cohort of patients with isolated CIED-related IE vs. left-sided valvular IE, using data from the NatIonal Danish endocarditis StUdieS (NIDUS) registry. A better understanding of these differences may be beneficial for optimizing diagnosis and guiding targeted preventive and treatment strategies.

## Methods

### Data source

This study was based on the NIDUS registry, which included all Danish citizens diagnosed with definite or possible IE episodes from 1 January 2016 to 31 December 2021 according to the modified Duke and European Society of Cardiology (ESC) 2015 diagnostic criteria. Each episode of IE during the study period was included, and patients with recurrent episodes of IE were registered multiple times. Detailed descriptions of the NIDUS registry’s design and data collection methodology have been published previously.^7,8^ The registry comprises detailed data on baseline characteristics, imaging [including echocardiography and positron emission tomography/computed tomography (PET/CT) scans], microbial aetiology, surgical treatment during hospitalization, antibiotic treatment, focus of infection, details on discharge, and clinical outcomes.

### Study population

**Figure 1:**
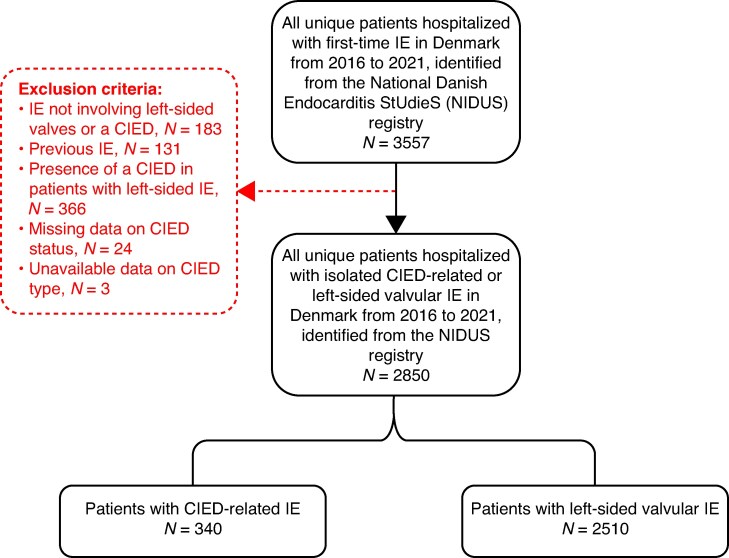
Flowchart of patient selection. CIED, cardiac implantable electronic device; IE, infective endocarditis.

The study population consisted of all patients diagnosed with a first-time IE episode in Denmark between 2016 and 2021, as recorded in the NIDUS registry. Patients were categorized into two mutually exclusive groups: (1) isolated CIED-related IE without concomitant valvular IE or (2) left-sided valvular IE. Patients with CIED-related IE had transvenous pacemakers (PMs), implantable cardioverter–defibrillators (ICDs), or cardiac resynchronization therapy devices (CRTs). We excluded patients with IE located at sites other than left-sided valves or a CIED (*n* = 183) and those with any previous episode of IE (*n* = 131). Additionally, we excluded patients with left-sided IE and a concomitant CIED, irrespective of CIED infection status (*n* = 366), patients with missing data on CIED status (*n* = 24), and patients with missing data on CIED type (*n* = 3) (*Figure 1*). These patients were excluded to allow comparison between two distinct clinical entities in the primary analysis.

### Microbial aetiology

Microbial aetiology was classified based on data from positive blood cultures, and in the case of multiple microorganisms, the most likely primary microbiological cause was identified. If blood cultures were negative, the aetiology was determined according to predefined criteria using polymerase chain reaction (PCR) results from excised valves, removed CIEDs, or other extracted relevant foreign material. Microbial aetiology was subsequently categorized as follows: *Staphylococcus aureus* (*S. aureus*), *Streptococcus* spp., *Enterococcus* spp., coagulase-negative staphylococci (CoNS), other microorganisms (including fungi and HACEK bacteria, i.e. *Haemophilus* spp., *Aggregatibacter actinomycetemcomitans*, *Cardiobacterium hominis*, *Eikenella corrodens*, and *Kingella kingae*), and culture-negative IE.

Both culture-negative and blood culture-negative IE were reported separately in the results.

### Study outcomes

The primary outcome was all-cause mortality within 6 months after IE diagnosis. The secondary outcomes were IE-related outcomes, which included (i) relapse of bacteraemia with the same microorganism, (ii) relapse of IE with the same microorganism, and (iii) reinfection (i.e. new IE episode with a different microorganism) within 6 months after IE discharge in patients discharged alive. These secondary outcomes were analysed both separately and as a composite outcome comprising all three.

For the primary outcome, patients were followed from IE diagnosis for up to 6 months until the first recorded occurrence of death, the date of last clinical follow-up, or the end of the study (31 December 2021). For secondary outcomes, patients were followed from the date of IE discharge for up to 6 months until the first recorded occurrence of an IE-related outcome, death, or the date of last clinical follow-up.

Study outcomes were compared between patients with isolated CIED-related and those with left-sided IE. In a subgroup analysis of patients with isolated CIED-related IE, outcomes were evaluated based on CIED removal status during IE admission.

### Statistical analyses

Categorical variables were reported as frequencies and percentages and continuous variables as medians with interquartile range (25th–75th percentiles). Differences between groups were assessed using the chi-squared test or Fisher’s exact test for categorical variables, as appropriate, and the Wilcoxon rank-sum test for continuous variables. When <5% of observations were missing, proportions were calculated based on available cases. For variables with ≥5% missing data, proportions were calculated using the total cohort as the denominator.

Kaplan–Meier estimates were used to calculate cumulative incidence of all-cause mortality, and differences between groups were evaluated using the log-rank test. The Aalen–Johansen estimator was used to calculate the cumulative incidence of IE-related outcomes, considering competing risk of all-cause mortality, and differences between groups were assessed using Gray’s test.

Multivariable Cox regression models were used to estimate adjusted hazard ratios (HRs) of all-cause mortality for isolated CIED-related IE vs. left-sided IE, as well as for CIED removal vs. no CIED removal. Results were reported as HRs with 95% confidence intervals (CIs). The models were adjusted for clinically relevant variables, including sex, age (continuous), microbial aetiology, active cancer, diabetes, kidney disease, liver disease, chronic obstructive pulmonary disease (COPD), and heart failure. Patients with missing data in one or more covariates were excluded from the multivariable Cox regression analyses by complete-case analysis, with <1.5% missing data in total. The model assumptions, including the proportional hazards assumption, absence of interaction between exposure and other clinically relevant covariates, and linearity of continuous variables, were met.

All statistical coding and analyses were performed using the SAS Enterprise Guide, version 8.4 (SAS Institute, Inc., Cary, NC, USA). A *P*-value <0.05 was considered as statistically significant.

### Sensitivity analyses

To assess whether exclusion of patients with left-sided IE and a CIED influenced the primary results, we compared baseline characteristics and outcomes between patients with isolated CIED-related IE and those with left-sided IE and a CIED, irrespective of concomitant CIED infection.

The primary multivariable Cox regression analysis of mortality was repeated including only patients with definite IE according to the modified Duke and ESC 2015 criteria.

The primary multivariable Cox regression model was further adjusted for factors of acute disease severity, including sepsis and ICU admission.

Finally, timing of CIED removal was evaluated using a 7-day landmark approach to mitigate immortal time bias. Patients who died within 7 days of diagnosis were excluded, and follow-up was initiated on Day 7. Early removal (≤7 days) was compared with no removal by Day 7, the latter including both delayed removal (>7 days) and no removal during admission.

### Ethics

The study was conducted in accordance with national ethical guidelines for registry-based studies issued by the Danish National Committee on Health Research Ethics. The requirement for informed consent was waived, and data collection was approved by the Danish Data Protection Agency (*P*-2020–92). Data with fewer than three observations were consistently reported as ‘<3’.

## Results

The NIDUS cohort comprised 3557 unique patients with first-time IE between 2016 and 2021. After applying the predefined exclusion criteria (*Figure 1*), including exclusion of patients with left-sided IE and a concomitant CIED (*n* = 366) and those with missing CIED status or CIED type (*n* = 27), the final study cohort comprised 340 (11.9%) patients with isolated CIED-related IE and 2510 (88.1%) patients with left-sided valvular IE.

### Baseline characteristics

Baseline characteristics, stratified by isolated CIED-related IE and left-sided IE, are presented in *Table 1*. Patients with CIED-related IE were older than those with left-sided IE, with a median age of 76.1 years (IQR: 68.2–81.7) vs. 73.2 years (IQR: 64.0–80.1), respectively (*P* < 0.001). The proportion of males was higher in patients with CIED-related IE compared with those with left-sided IE (78.5% vs. 65.9%) (*P* < 0.001). Diabetes was more common in CIED-related IE (32.9% vs. 21.9%) (*P* < 0.001), whereas dialysis was less common (2.1% vs. 5.3%) (*P* = 0.009) compared with left-sided IE. The duration of hospital admission was comparable between the two groups. Length of antibiotic treatment was shorter in patients with CIED-related IE compared with those with left-sided IE (31 days [IQR: 20–43] vs. 38 days [IQR: 28–45], *P* < 0.001). The presence of fever at admission >38 degrees Celsius was lower in patients with CIED-related IE than in those with left-sided IE (55.4% vs. 61.5%) (*P* = 0.03). Among patients with left-sided IE, 589 (23.5%) underwent valve surgery during admission.

**Table 1:** Baseline characteristics in patients with CIED-related and left-sided infective endocarditis

Variable	Left-sided IE, *n* = 2510 (%)	CIED-related IE, *n* = 340 (%)	*P*-value
**Demographics**			
Sex (Male)	1655 (65.9)	267 (78.5)	<0.001
Age [median (IQR)], years	73.2 [64.0, 80.1]	76.1 [68.2, 81.7]	<0.001
**Age group**			
<70 years	987 (39.3)	94 (27.7)	<0.001
70–80 years	889 (35.4)	141 (41.5)	
>80 years	634 (25.3)	105 (30.9)	
**Prosthetic valve endocarditis**	527 (21.0)	0 (0.0)	<0.001
**Definite IE according to the modified Duke/ESC 2015 criteria**	2032 (81.0)	250 (73.5)	0.001
**Length of admission, days [median (IQR)]**	35 [25, 47]	35 [25, 45]	0.46
**Duration of disease before diagnosis of endocarditis, days [median (IQR)]**	8 [4, 21]	7 [3, 16]	0.02
**Length of antibiotic treatment, days [median (IQR)]**	38 [28, 45]	31 [20, 43]	<0.001
**PET-CT**	1690 (67.4)	220 (65.1)	0.39
**Type of diagnostic echocardiography**			
TTE	180 (7.2)	24 (7.1)	0.94
TEE	2330 (92.8)	316 (92.9)	
**History of comorbidity**			
Prior heart failure	292 (11.7)	156 (46.2)	<0.001
Diabetes	549 (21.9)	112 (32.9)	<0.001
Kidney disease	401 (16.0)	73 (21.5)	0.01
Dialysis	133 (5.3)	7 (2.1)	0.009
Liver disease	127 (5.1)	6 (1.8)	0.007
COPD	365 (14.6)	54 (15.9)	0.53
Previous cancer	356 (14.2)	51 (15.0)	0.71
Active cancer	230 (9.2)	22 (6.5)	0.10
Congenital heart disease	93 (3.7)	6 (1.8)	0.066
Native valvular heart disease	349 (13.9)	43 (13.0)	0.63
Stroke	344 (13.7)	54 (16.0)	0.27
**Pharmacotherapy within 3 months prior to admission**			
Antithrombotic drugs	1334 (53.7)	256 (76.0)	<0.001
Immunosuppressant drugs	282 (11.4)	36 (10.7)	0.70
**Symptoms**			
Fever at admission (>38 degrees)	1480 (61.5)	184 (55.4)	0.03
Myalgia at admission^a^	654 (26.1)	78 (22.9)	0.13
Dyspnoea at admission	760 (31.8)	112 (33.7)	0.48
Significant weight loss at admission (>5 kg)^a^	354 (14.1)	37 (10.9)	0.091
**Weight (kg) [median (IQR)]** ^a^	78 [67, 90]	80 [69.8, 93.0]	0.03
**Dental procedure 3 months prior to admission**	115 (4.7)	11 (3.4)	0.26
**Active alcohol consumption** ^a^	1675 (66.7)	225 (66.2)	0.71
**Active smoking** ^a^	490 (19.5)	46 (13.5)	0.01
**Active intravenous (IV) drug abuse**	48 (2.0)	0 (0.0)	0.01
**Admission to a highly specialized centre** ^a^	1655 (65.9)	291 (85.6)	<0.001
**Self-reliant at activities of daily living**	1801 (72.5)	228 (67.9)	0.077
**Type of aid**			
Dependent on accessibility aid	202 (8.2)	30 (8.9)	0.31
Domiciliary care	378 (15.3)	62 (18.5)	
Nursing home	98 (4.0)	16 (4.8)	
**Retired**	1914 (77.2)	295 (87.3)	<0.001
**Previous heart valve surgery**	610 (24.3)	52 (15.3)	<0.001
**IE-related complications at admission**			
Heart failure	65 (2.6)	16 (4.7)	0.03
Sepsis	595 (23.7)	70 (20.6)	0.20
Intensive care unit admission	522 (20.8)	50 (14.7)	0.008

^a^ ≥ 5% missing data. For variables with ≥ 5% missing data, proportions were calculated using the total column as the denominator.

CIED, cardiac implantable electronic device; COPD, chronic obstructive pulmonary disease; IE, infective endocarditis

### CIED-related infective endocarditis and practice patterns of CIED removal

In patients with CIED-related IE, the majority had PMs (59.1%), followed by ICDs (28.5%) and CRTs (12.4%). Cardiac implantable electronic device removal during IE admission was performed in 266 (78.2%). Baseline characteristics stratified by CIED removal status are summarized in Supplementary material online, *Table S1*. Patients who did not undergo CIED removal were older (*P* < 0.001), with 54.1% being more than 80 years of age. *Enterococcus* spp. was more than twice as prevalent in patients who did not undergo CIED removal compared with those who did undergo removal (*P* < 0.001). The prevalence of individual comorbidities was similar between groups (*P* > 0.05). Patients who were self-reliant in activities of daily living comprised 45.9% of the non-removal group and 74.0% of the removal group (*P* < 0.001). Overall, 16.2% of patients who did not undergo CIED removal were nursing home residents, compared with 1.5% of those who underwent removal.

Among patients who did not undergo CIED removal (*n* = 74), 15 (20.3%) were discharged alive on chronic suppressive antibiotic therapy, and 43 (58.1%) were discharged alive without such therapy, while 16 (21.6%) died during hospitalization. According to the diagnostic criteria, 24.3% of patients who did not undergo CIED removal had possible IE, compared with 27.1% of those who underwent removal.

### Microbial aetiology

**Figure 2:**
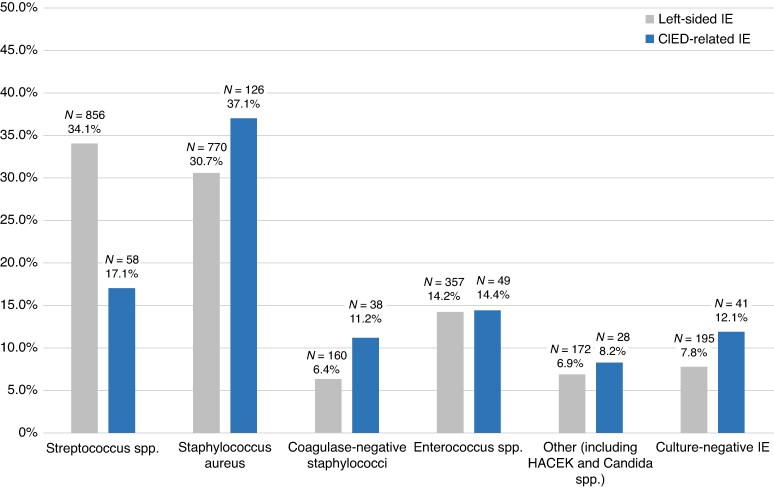
Distribution of microbial aetiology in patients with CIED-related and left-sided infective endocarditis. CIED, cardiac implantable electronic device; IE, infective endocarditis.

The distribution of microbial aetiology is illustrated in *Figure 2* for patients with CIED-related IE and for those with left-sided IE. Overall, microbial aetiology differed between the two groups (*P* < 0.001). *Staphylococcus aureus* was the most common pathogen in patients with CIED-related IE (37.1% vs. 30.7%), whereas *Streptococcus* spp. was the most common pathogen in patients with left-sided IE (34.1%), representing a twofold higher prevalence than in patients with CIED-related IE (17.1%). The prevalence of CoNS was higher in CIED-related IE than in left-sided IE, while the prevalence of *Enterococcus* spp. was comparable between both groups. Culture-negative IE (based on blood cultures and cultures of material extracted from valves or CIEDs) was observed in 12.1% of patients with CIED-related IE and 7.8% of those with left-sided IE, whereas truly blood culture-negative IE was found in 18.4% and 8.9%, respectively.

**Figure 3:**
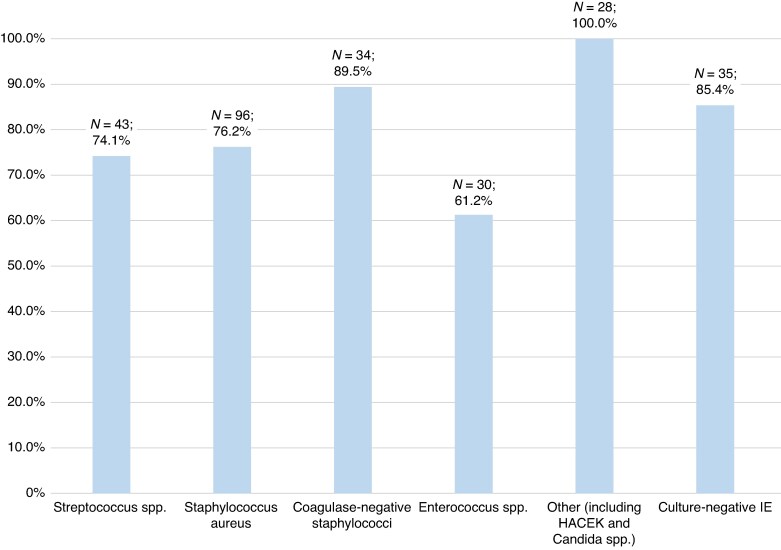
Frequency of CIED removal according to microbial aetiology. CIED, cardiac implantable electronic device; IE, infective endocarditis.

In a subgroup analysis of patients with CIED-related IE (*Figure 3*), the prevalence of CIED removal was 100% in patients with other aetiologies (including HACEK bacteria and fungi), 89.5% in patients with CoNS, 76.2% in patients with *Staphylococcus aureus*, and 85.4% in patients with culture-negative IE. Patients with *Enterococcus* spp. had the lowest prevalence of CIED removal (61.2%).

### All-cause mortality

**Figure 4:**
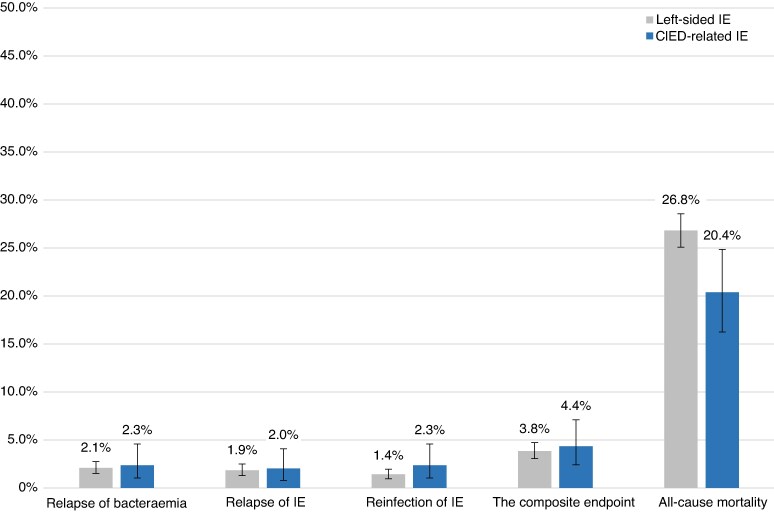
Six-month cumulative incidence of relapse of bacteraemia, relapse of IE, reinfection of IE, the composite outcome, and all-cause mortality in patients with CIED-related and left-sided infective endocarditis. Relapse of bacteraemia and relapse of IE were defined as infection with the same microorganism, whereas reinfection of IE was defined as infection with a different microorganism. The composite outcome included all three, with death as a competing risk. CIED, cardiac implantable electronic device; IE, infective endocarditis.

In-hospital mortality was 12.1% in patients with CIED-related IE and 17.9% in those with left-sided IE (*P* = 0.007). The 6-month cumulative incidence of all-cause mortality after IE diagnosis was 20.4% (95% CI: 16.2–24.9%) in patients with CIED-related IE and 26.8% (95% CI: 25.1–28.6%) in those with left-sided IE (*P* = 0.009) (*Figure 4* and Supplementary material online, *Figure S1*). In both unadjusted and adjusted analyses, CIED-related IE was associated with a lower 6-month rate of all-cause mortality compared with left-sided IE (unadjusted HR: 0.72 [95% CI: 0.56–0.92], *P* = 0.009; adjusted HR: 0.52 [95% CI: 0.40–0.68], *P* < 0.001).

**Figure 5:**
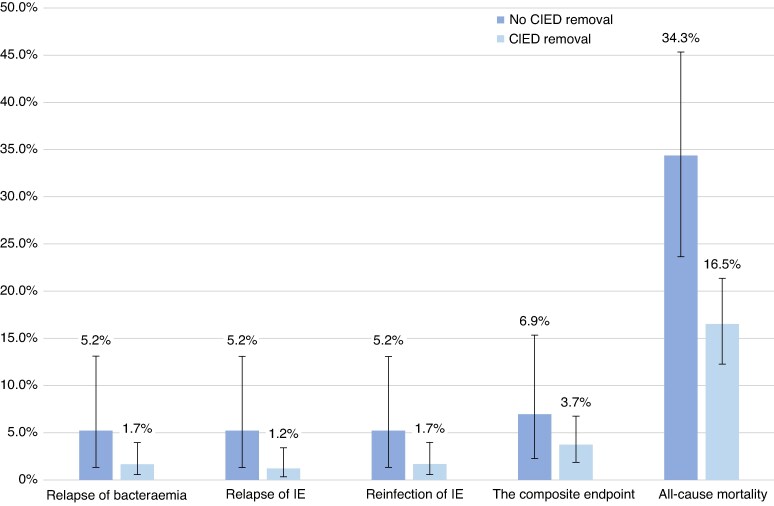
Six-month cumulative incidence of relapse of bacteraemia, relapse of IE, reinfection of IE, the composite outcome, and all-cause mortality in patients with CIED-related IE according to CIED removal status. Relapse of bacteraemia and relapse of IE were defined as infection with the same microorganism, whereas reinfection of IE was defined as infection with a different microorganism. The composite outcome included all three, with death as a competing risk. CIED, cardiac implantable electronic device; IE, infective endocarditis.

Among patients with CIED-related IE, in-hospital mortality was 21.6% in those who did not undergo CIED removal and 9.4% in those who underwent removal (*P* = 0.004). The 6-month cumulative incidence of all-cause mortality after IE diagnosis was 34.3% (95% CI: 23.6–45.3%) in the non-removal group and 16.5% (95% CI: 12.3–21.3%) in the removal group (*P* < 0.001) (*Figure 5*). Cardiac implantable electronic device removal was associated with a significantly lower 6-month rate of all-cause mortality compared with no removal (unadjusted HR: 0.41 [95% CI: 0.25–0.67], *P* < 0.001; adjusted HR: 0.44 [95% CI: 0.25–0.76], *P* = 0.004).

### Infective endocarditis-related outcomes

The 6-month cumulative incidences of IE-related outcomes for patients with CIED-related IE and left-sided IE following discharge are shown in *Figure 4*. The 6-month cumulative incidences in patients with CIED-related IE vs. left-sided IE were 2.3% (95% CI: 1.0–4.6%) vs. 2.1% (95% CI: 1.5–2.8%) for relapse of bacteraemia (*P* = 0.78), 2.0% (95% CI: 0.83–4.1%) vs. 1.9% (95% CI: 1.3–2.5%) for relapse of IE (*P* = 0.85), 2.3% (95% CI: 1.0–4.6%) vs. 1.4% (95% CI: 0.97–2.0%) for reinfection of IE (*P* = 0.22), and 4.4% (95% CI: 2.4–7.1%) vs. 3.8% (95% CI: 3.1–4.7%) for the composite endpoint of all three (*P* = 0.67). In a subgroup analysis of patients with CIED-related IE stratified by CIED removal status (*Figure 5*), the 6-month cumulative incidences of relapse of bacteraemia (*P* = 0.11), relapse of IE (*P* = 0.055), reinfection of IE (*P* = 0.11), and the composite endpoint (*P* = 0.29) were similar between groups.

### Sensitivity analyses

In a sensitivity analysis, 366 patients were identified with left-sided IE and a CIED, of whom 143 (39.1%) had concomitant CIED infection, whereas 223 (60.9%) had a bystander CIED. Cardiac implantable electronic device removal was performed in 39.9% of the overall cohort, 65.0% of patients with concomitant CIED infection, and 23.8% of those with a bystander CIED.

The median age was 75.9 years (IQR 70.3–82.9) in patients with concomitant CIED infection compared with 80.3 years (IQR 74.7–84.8) in those with a bystander CIED.

Patients with left-sided IE and a CIED, irrespective of CIED infection status, differed in several baseline characteristics compared with those with isolated CIED-related IE (see Supplementary material online, *Table S2*), and they also differed from patients with left-sided IE without a CIED. Compared with patients with CIED-related IE, those with left-sided IE and a CIED were older, had longer hospital admissions and antibiotic treatment, were more often dependent on accessibility aids, were more frequently nursing home residents, and had a higher prevalence of *Streptococcus* spp. and *Enterococcus* spp. (all *P* < 0.05). The prevalence of individual comorbidities, fever, myalgia, and dyspnoea was similar between groups (all *P* > 0.05) (see Supplementary material online, *Table S2*).

The 6-month cumulative incidences of relapse of bacteraemia (*P* = 0.82), relapse of IE (*P* = 0.95), reinfection of IE (*P* = 0.96), and the composite endpoint (*P* = 0.79) did not differ significantly between the groups, whereas mortality was significantly higher in patients with left-sided IE and a CIED than in those with CIED-related IE (*P* < 0.001), consistent with the primary results (see Supplementary material online, *Figure S2*). CIED-related IE remained associated with lower 6-month all-cause mortality compared with left-sided IE with a CIED (unadjusted HR: 0.53 [95% CI: 0.39–0.71], *P* < 0.001; adjusted HR: 0.56 [95% CI: 0.41–0.76], *P* < 0.001).

In a sensitivity analysis restricted to patients with definite IE, CIED-related IE remained associated with lower 6-month mortality in adjusted analyses (adjusted HR: 0.53 [95% CI: 0.39–0.72]).

In an additional sensitivity analysis further adjusting for sepsis and ICU admission, the association between CIED-related IE and lower 6-month mortality remained unchanged (adjusted HR: 0.52 [95% CI: 0.40–0.69]).

The median time from IE diagnosis to CIED removal was 4 days (IQR: 1–7). In a 7-day landmark analysis, including patients alive on Day 7, the cumulative incidence of mortality did not differ significantly between patients with early CIED removal (≤7 days) (*n* = 208) and those without removal by Day 7 (*n* = 126) (*P* = 0.082) (see Supplementary material online, *Figure S3*). Among the latter, 56 patients underwent delayed removal (>7 days). In adjusted Cox regression analyses, early removal was not independently associated with mortality (adjusted HR: 0.67 [95% CI: 0.40–1.11]).

## Discussion

In this nationwide study of patients with isolated CIED-related IE and left-sided IE, we identified four major findings. First, patients with CIED-related IE were older, had a higher proportion of males, and were less likely to present with fever, when compared to left-sided IE. Second, staphylococcal bacteraemia and culture-negative IE were more prevalent in patients with CIED-related IE. Third, although the 6-month cumulative incidences of IE-related outcomes were similar between groups, patients with CIED-related IE had lower all-cause mortality. Fourth, CIED removal was performed in most patients with CIED-related IE (*Graphical Abstract*).

### Differences in baseline characteristics

Patients with CIED-related IE were older, more often male, and less likely to present with fever at admission. These demographic differences are consistent with previous studies, where advanced age and male predominance have been consistently associated with CIED infection,^1,3,9,10^ including in comparison with left-sided IE.^6^

The lower presence of fever among patients with CIED-related IE compared with those with left-sided IE is an important finding, in line with a previous study.^6^ Fever is a cardinal manifestation of IE and plays a significant role in early diagnosis.^4,5,11,12^ However, CIED-related IE, particularly in the absence of pocket infection, may be challenging to diagnose, presenting with atypical or non-specific symptoms, in which fever may be the only clinical manifestation.^5,11,13^ Advanced age and comorbidities such as diabetes may all attenuate the systemic inflammatory responses, leading to less frequent fever and delayed diagnosis in patients with CIED-related IE.^14,15^ Our findings reinforce the importance of increased diagnostic awareness in elderly patients with CIEDs who present with non-specific symptoms, even in the absence of fever, and highlight the need for a low threshold for obtaining blood cultures.

Furthermore, we observed a higher prevalence of diabetes in patients with CIED-related IE, in line with previously identified risk factors for CIED infection.^3,14–16^ Diabetes is associated with impaired host immunity and delayed wound healing, which may predispose to CIED infection.^17,18^ In contrast, the lower prevalence of dialysis treatment in this group may reflect different comorbidity patterns compared with patients with left-sided IE.^3,19^ Patients on chronic dialysis more frequently undergo vascular access procedures and develop valvular calcification,^20,21^ both of which may predispose more strongly to left-sided IE than to CIED-related IE.^22^

### Microbiology

*S. aureus*, CoNs, and culture-negative IE were more common in patients with CIED-related IE. These findings are consistent with previous literature, which have identified staphylococci as the primary microbiological causes of CIED-related IE.^10,23–28^ These bacteria are more likely to form biofilms and adhere to nonbiological material such as CIED surfaces than other microorganisms.^25,29,30^ In accordance with the current treatment guidelines, complete CIED removal should be considered in cases of bacteraemia or fungaemia caused by *S. aureus*, CoNS, *Cutibacterium* spp., or *Candida* spp.^4,5^

In addition, culture-negative IE was more prevalent among patients with CIED-related IE. This may be explained by infection localized to the device pocket, by prior antibiotic treatment, or by the presence of fastidious pathogens or biofilm-associated pathogens that are undetected by conventional culture techniques.^4,5,31,32^ In this study, the proportion of truly blood culture-negative IE was initially 18% in patients with CIED-related IE, but this was reduced to 12% after reclassification based on cultures from extracted valves and CIEDs. These findings reinforce current guideline recommendations to obtain multiple blood cultures prior to initiating antibiotic therapy and to perform systematic sampling of pocket and lead material during removal to optimize identification of the causative pathogen.^4,5,11,12^

### Clinical outcomes

In this nationwide cohort, the rates of IE-related outcomes such as relapse and reinfection were low and did not differ significantly between patients with CIED-related IE and those with left-sided IE. We observed a lower risk of all-cause mortality in patients with CIED-related IE, consistent with findings from recent registry-based studies^1,3,33^ and a previous single-centre study, which reported an in-hospital mortality of 28% in CIED-related IE and 35% in left-sided IE, although the difference was not statistically significant.^6^ However, despite this relative difference, the absolute 6-month mortality of 20% in CIED-related IE in our study remains substantial, underscoring the serious prognosis associated with this condition. Overall, these findings indicate that outcomes may depend on factors such as patient selection, clinical management, and CIED removal practices.^14,34–36^ Patients with CIED-related IE may be diagnosed earlier than those with left-sided IE, as having a CIED often requires diagnostic imaging when infection is suspected.^4,5,11,34^ However, because CIED-related IE may present with fewer systemic symptoms such as fever, the initial clinical suspicion can be delayed. Furthermore, the high prevalence of CIED removal in our cohort may contribute to better survival, consistent with current literature and guidelines suggesting that complete CIED removal is associated with improved outcomes.^5,11,33–38^ The relatively low prevalence of valve surgery among patients with left-sided IE may reflect the advanced age and comorbidity burden in this nationwide cohort, which may have limited surgical eligibility and potentially contributed to the higher mortality observed in this group. These findings underscore differences in prognosis between CIED-related and left-sided IE. In sensitivity analyses including patients with left-sided IE and a concomitant CIED as an additional comparison group, the overall findings remained consistent. This subgroup represents a clinically relevant and heterogeneous population in real-world practice. Further research is warranted to determine whether earlier diagnosis, broader use of advanced microbiological techniques, and optimized management strategies may improve outcomes across these patient groups.

### Management of CIED-related IE

In this study, CIED removal was performed in the majority of patients with CIED-related IE, consistent with current guidelines that recommend complete CIED removal.^4,5,11,34^ According to current literature, CIED removal is associated with improved outcomes, while conservative management with antibiotic treatment alone is associated with high risk of relapse and mortality.^5,11,33–38^ Nevertheless, some patients in our cohort did not undergo CIED removal. Among these, 20% were discharged alive on chronic suppressive antibiotic therapy, 58% were discharged without such treatment, and the remaining 22% died during admission. These findings reflect the heterogeneity in clinical practice and suggest that advanced age, frailty, or procedural risk may influence decision-making when removal is considered not feasible.^33,35–37,39^ In addition, delayed or omitted CIED removal has been associated with worse clinical outcomes and increased healthcare utilization and expenditures.^40^ Furthermore, admission to centres with higher lead extraction volumes may influence the likelihood of CIED removal, reflecting the importance of specialized expertise in the management of CIED-related IE.^41^

We also observed that the frequency of CIED removal varied by microbial aetiology. Notably, all patients with fungal infections underwent CIED removal, reflecting both the severity of these infections and the limited possibility of infection eradication without CIED removal.^4,5^ In contrast, CIED removal was least frequent among patients with *Enterococcus* spp. (61%), likely due to advanced age, multimorbidity, and frequent polymicrobial aetiology.^4,42,43^ These findings emphasize the persistent gap between guideline recommendations and real-world practice, underscoring the need for careful risk–benefit assessment and multidisciplinary decision-making to optimize outcomes for patients with CIED-related IE.

### Strengths and limitations

This study provides complete, nationwide data on unselected patients with IE, with no loss to follow-up, representing the main strength of the NIDUS registry. Nevertheless, several limitations should be acknowledged. Patients were included only if admitted with an ICD-10 diagnosis of IE. Thus, some cases may have been missed, particularly among patients who died before confirmation of IE diagnosis. Data collection was performed by a large group of healthcare professionals, which may have introduced minor interobserver variability. However, this was examined, and interrater assessments demonstrated high concordance for key variables.^7,8^

Furthermore, information bias cannot be excluded, as the prevalence of certain clinical findings, such as symptom presentation and lifestyle factors, may be systematically underestimated due to underreporting in medical records. Several important clinical variables, including laboratory data, serial blood cultures, and lead vegetation size, were not available.

The observational design of the study limits conclusions to associations rather than causality. Although multivariable Cox regression analyses were adjusted for potential confounders, residual confounding remains possible. In addition, confounding by indication may have influenced findings in patients with CIED-related IE stratified by CIED removal status, as those who did not undergo removal were older and frailer and had higher mortality risk.

Finally, patients with left-sided IE and a CIED were excluded from the main study population to maintain a clinically coherent comparison between isolated CIED-related IE and left-sided valvular IE without a CIED. These patients represented an older and frailer subgroup, and their inclusion would have introduced substantial heterogeneity. Notably, they exhibited higher mortality than both primary groups, while IE-related outcomes were similar, indicating that their exclusion was conservative and supporting the robustness of our findings.

## Conclusions

In this nationwide study, patients with isolated CIED-related IE differed from those with left-sided IE. *Staphylococcus* species were more common in patients with CIED-related IE, and despite higher age and differences in comorbidities, mortality was lower, while IE-related outcomes were similar. These findings suggest that isolated CIED-related IE represents a distinct clinical entity and may help inform more focused diagnostic and preventive strategies in this population.

## Supplementary Material

euag075_Supplementary_Data

## Data Availability

The data underlying this study cannot be shared publicly owing to data protection requirements.
