# Supplementary material for: Clinical characteristics, treatment, and outcomes in CIED-related vs. left-sided infective endocarditis: a nationwide study from the NIDUS registry
Source: Europace. 2026 Apr 9;28(4):euag075. doi: 10.1093/europace/euag075 (PMC13126043; doi:10.1093/europace/euag075)
Supplement: euag075_Supplementary_Data [file euag075_supplementary_data.docx]

**Supplementary data**

**Alhakak et al.**

**Title: Clinical characteristics, treatment, and outcomes in CIED-related versus left-sided infective endocarditis: a nationwide study from the NIDUS registry**

**Table S1: Baseline characteristics in patients with CIED-related IE according to CIED removal status.**

| **Variable** | **No CIED removal (N= 74)** | **CIED removal (N=266)** | **P-value** |
| --- | --- | --- | --- |
| **Demographics** |  |  |  |
| Sex (Male) | 57(77.0) | 210(78.9) | 0.72 |
| Age (median [IQR]), years | 80.9 [74.2, 85.7] | 74.6 [66.5, 79.7] | <0.001 |
| **Age group** |  |  |  |
| <70 years | 8 (10.8) | 86 (32.3) | <0.001 |
| 70-80 years | 26(35.1) | 115(43.2) |  |
| >80 years | 40(54.1) | 65(24.4) |  |
| **CIED type** |  |  |  |
| Pacemaker | 47 (63.5) | 154 (57.9) | 0.68 |
| Implantable cardioverter-defibrillator | 19 (25.7) | 78 (29.3) |  |
| Cardiac resynchronization therapy | 8 (10.8) | 34 (12.8) |  |
| **Possible or definite IE according to the modified Duke/ESC 2015 criteria** |  |  |  |
| Definite | 56 (75.7) | 194 (72.9) | 0.64 |
| Possible | 18(24.3) | 72(27.1) |  |
| **Length of admission, days (median [IQR])** | 33 [18, 48] | 35 [26, 44] | 0.34 |
| **Duration of disease before diagnosis of endocarditis, days (median [IQR])** | 6 [ 3.0, 15.0] | 7.5 [ 4.0, 16.5] | 0.095 |
| **Length of antibiotic treatment, days (median [IQR])** | 33.5 [16.0, 58.0] | 30 [20.0, 42.0] | 0.37 |
| **PET-CT** | 45(60.8) | 175(66.3) | 0.38 |
| **Type of diagnostic echocardiography** |  |  |  |
| TTE | 9 (12.2) | 15 (5.6) |  |
| TEE | 65(87.8) | 251(94.4) | 0.053 |
| **Microbial aetiology** |  |  |  |
| *Streptococcus spp.* | 15(20.3) | 43(16.2) | <0.001 |
| *Staphylococcus aureus* | 30(40.5) | 96(36.1) |  |
| Coagulase-negative staphylococci | 4(5.4) | 34(12.8) |  |
| *Enterococcus spp.* | 19(25.7) | 30(11.3) |  |
| Other microbial aetiology^a^ | 0(0.0) | 28(10.5) |  |
| Culture-negative | 6(8.1) | 35(13.2) |  |
| **Ejection fraction levels at diagnostic echocardiography**** |  |  |  |
| Normal | 26 (35.1) | 97 (36.5) | 0.76 |
| Mild reduction | 13(17.6) | 43(16.2) |  |
| Moderate reduction | 11(14.9) | 58(21.8) |  |
| Severe reduction | 8(10.8) | 33(12.4) |  |
| **History of comorbidity** |  |  |  |
| Prior heart failure | 28(38.4) | 128(48.3) | 0.13 |
| Diabetes | 28(37.8) | 84(31.6) | 0.31 |
| Kidney disease | 17(23.0) | 56(21.1) | 0.73 |
| Dialysis | <3 | 6(2.3) | – |
| Liver disease | <3 | 4(1.5) | – |
| COPD | 8(10.8) | 46(17.3) | 0.18 |
| Previous cancer | 13(17.6) | 38(14.3) | 0.48 |
| Active cancer | 8(11.0) | 14(5.3) | 0.080 |
| Congenital heart disease | <3 | 5(1.9) | – |
| Stroke | 15(20.3) | 39(14.8) | 0.25 |
| **Pharmacotherapy within 3 months prior to admission** |  |  |  |
| Anti thrombotic drugs | 60(81.1) | 196(74.5) | 0.24 |
| Immunosuppressant drugs | 11(15.1) | 25(9.5) | 0.17 |
| **Previous heart valve surgery** | 18(24.3) | 34(12.8) | 0.01 |
| **Symptoms** |  |  |  |
| Fever at admission (>38 degrees) | 37(50.0) | 147(57.0) | 0.29 |
| Myalgia at admission | 21(28.4) | 57(22.2) | 0.27 |
| Dyspnoea at admission | 33(44.6) | 79(30.6) | 0.03 |
| Significant weight loss at admission (>5 kg)* | 8(10.8) | 29(10.9) | 0.90 |
| **Weight (kg) (median [IQR])*** | 76 [65.5, 87.0] | 81 [70.2, 95.0] | 0.02 |
| **Dental procedure 3 months prior to admission** | <3 | 9(3.5) | – |
| **Active alcohol consumption*** | 44(59.5) | 181(68.0) | 0.29 |
| **Active smoking*** | 13(17.6) | 33(12.4) | 0.15 |
| **Admission to a highly specialised centre*** | 40 (54.1) | 251 (94.4) | <0.001 |
| **Self-reliant at activities of daily living** | 34(45.9) | 194(74.0) | <0.001 |
| **Type of aid** |  |  |  |
| Dependent on accessibility aid | 6 (8.1) | 24 (9.2) | <0.001 |
| Domiciliary care | 22(29.7) | 40(15.3) |  |
| Nursing home | 12(16.2) | 4(1.5) |  |
| **Retired** | 73(98.6) | 222(84.1) | <0.001 |
| **IE-related complications at admission** |  |  |  |
| Heart failure | 4 (5.4) | 12 (4.5) | 0.75 |
| Sepsis at admission | 21(28.4) | 49(18.4) | 0.061 |
| Intensive care unit admission | 11(14.9) | 39(14.7) | 0.97 |

* ≥ 5% missing data; ** ≥ 10% missing data. For variables with ≥ 5% missing data, proportions were calculated using the total column as the denominator.

^a^Other microbial aetiology includes HACEK and *Candida* species.

Abbreviations: CIED, cardiac implantable electronic device; COPD, chronic obstructive pulmonary disease; IE, infective endocarditis

**Table S2: Baseline characteristics in patients with CIED-related and left-sided infective endocarditis with a CIED**

| Variable | **Left-sided IE and a CIED, N=366 (%)** | **CIED-related IE, N= 340 (%)** | **P-value** |
| --- | --- | --- | --- |
| **Demographics** |  |  |  |
| Sex (Male) | 257(70.2) | 267(78.5) | 0.01 |
| Age (median [IQR]), years | 79.0 [72.6, 84.4] | 76.1 [68.2, 81.7] | <0.001 |
| **Age group** |  |  |  |
| <70 years | 63 (17.2) | 94 (27.7) | <0.001 |
| 70-80 years | 141(38.5) | 141(41.5) |  |
| >80 years | 162(44.3) | 105(30.9) |  |
| **CIED type** |  |  |  |
| Pacemaker | 272 (76.0) | 201 (59.1) | <0.001 |
| Implantable cardioverter-defibrillator | 67 (18.7) | 97 (28.5) |  |
| Cardiac resynchronization therapy | 19 (5.3) | 42 (12.4) |  |
| **Possible or definite IE according to the modified Duke/ESC 2015 criteria** |  |  |  |
| Definite | 294 (80.3) | 250 (73.5) | 0.03 |
| Possible | 72(19.7) | 90(26.5) |  |
| **Length of admission (median [IQR])** | 40 [26, 49] | 35 [25, 45] | 0.02 |
| **Duration of disease before diagnosis of endocarditis (median [IQR])** | 8 [ 4, 19] | 7 [ 3, 16] | 0.23 |
| **Length of antibiotic treatment (median [IQR])** | 42 [27, 46] | 31 [20, 43] | <0.001 |
| **Microbial aetiology** |  |  |  |
| *Streptococcus* spp. | 95(26.0) | 58(17.1) | <0.001 |
| *Staphylococcus aureus* | 104(28.4) | 126(37.1) |  |
| Coagulase-negative staphylococci | 40(10.9) | 38(11.2) |  |
| *Enterococcus* spp. | 86(23.5) | 49(14.4) |  |
| Other microbial aetiology^a^ | 22(6.0) | 28(8.2) |  |
| Culture-negative | 19(5.2) | 41(12.1) |  |
| **PET-CT** | 245(66.9) | 220(65.1) | 0.60 |
| **Type of diagnostic echocardiography** |  |  |  |
| TTE | 20 (5.5) | 24 (7.1) | 0.38 |
| TEE | 346(94.5) | 316(92.9) |  |
| **Ejection fraction levels at baseline echocardiography**** |  |  |  |
| Normal | 133 (36.3) | 123 (36.2) | 0.083 |
| Mild reduction | 81(22.1) | 56(16.5) |  |
| Moderate reduction | 68(18.6) | 69(20.3) |  |
| Severe reduction | 28(7.7) | 41(12.1) |  |
| **History of comorbidity** |  |  |  |
| Prior heart failure | 147(40.5) | 156(46.2) | 0.13 |
| Diabetes | 112(30.6) | 112(32.9) | 0.50 |
| Kidney disease | 78(21.3) | 73(21.5) | 0.94 |
| Dialysis | 18(4.9) | 7(2.1) | 0.04 |
| Liver disease | 14(3.8) | 6(1.8) | 0.099 |
| COPD | 61(16.7) | 54(15.9) | 0.78 |
| Previous cancer | 64(17.5) | 51(15.0) | 0.36 |
| Active cancer | 32(8.7) | 22(6.5) | 0.26 |
| Congenital heart disease | 12(3.3) | 6(1.8) | 0.19 |
| Native valvular heart disease | 47(13.0) | 43(13.0) | 0.98 |
| Stroke | 74(20.2) | 54(16.0) | 0.14 |
| **Pharmacotherapy within 3 months prior to admission** |  |  |  |
| Anti thrombotic drugs | 302(83.2) | 256(76.0) | 0.02 |
| Immunosuppressant drugs | 38(10.5) | 36(10.7) | 0.93 |
| **Symptoms** |  |  |  |
| Fever at admission (>38 degrees) | 197(55.2) | 184(55.4) | 0.95 |
| Myalgia at admission | 86(24.2) | 78(23.6) | 0.84 |
| Dyspnoea at admission | 141(39.5) | 112(33.7) | 0.12 |
| Significant weight loss at admission (>5 kg)* | 53(14.5) | 37(10.9) | 0.16 |
| **Weight (kg) (median [IQR]) *** | 79.3 [68.0, 91.1] | 80.0 [69.8, 93.0] | 0.28 |
| **Dental procedure 3 months prior to admission** | 11(3.1) | 11(3.4) | 0.87 |
| **Active alcohol consumption*** | 227(62.0) | 225(66.2) | 0.083 |
| **Active smoking*** | 53(14.5) | 46(13.5) | 0.69 |
| **Admission to a highly specialized center*** | 253(69.1) | 291(85.6) | <0.001 |
| **Self-reliant at activities of daily living** | 201(55.2) | 228(67.9) | <0.001 |
| **Type of aid** |  |  |  |
| Dependent on accessibility aid | 59 (16.2) | 30 (8.9) | 0.002 |
| Domiciliary care | 76(20.9) | 62(18.5) |  |
| Nursing home | 28(7.7) | 16(4.8) |  |
| **Retired** | 330(90.4) | 295(87.3) | 0.19 |
| **Previous heart valve surgery** | 163(44.5) | 52(15.3) | <0.001 |
| **IE-related complications at admission** |  |  |  |
| Heart failure | 9(2.5) | 16(4.7) | 0.11 |
| Sepsis | 80(21.9) | 70(20.6) | 0.68 |
| Intensive care unit admission | 54(14.9) | 50(14.7) | 0.95 |

* ≥ 5% missing data; ** ≥ 10% missing data. For variables with ≥ 5% missing data, proportions were calculated using the total column as the denominator.

^a^Other microbial aetiology includes HACEK and *Candida* species.

Abbreviations: CIED, cardiac implantable electronic device; COPD, chronic obstructive pulmonary disease; IE, infective endocarditis

**Figure S1: Six-month cumulative incidence of all-cause mortality in patients with CIED-related and left-sided infective endocarditis.**


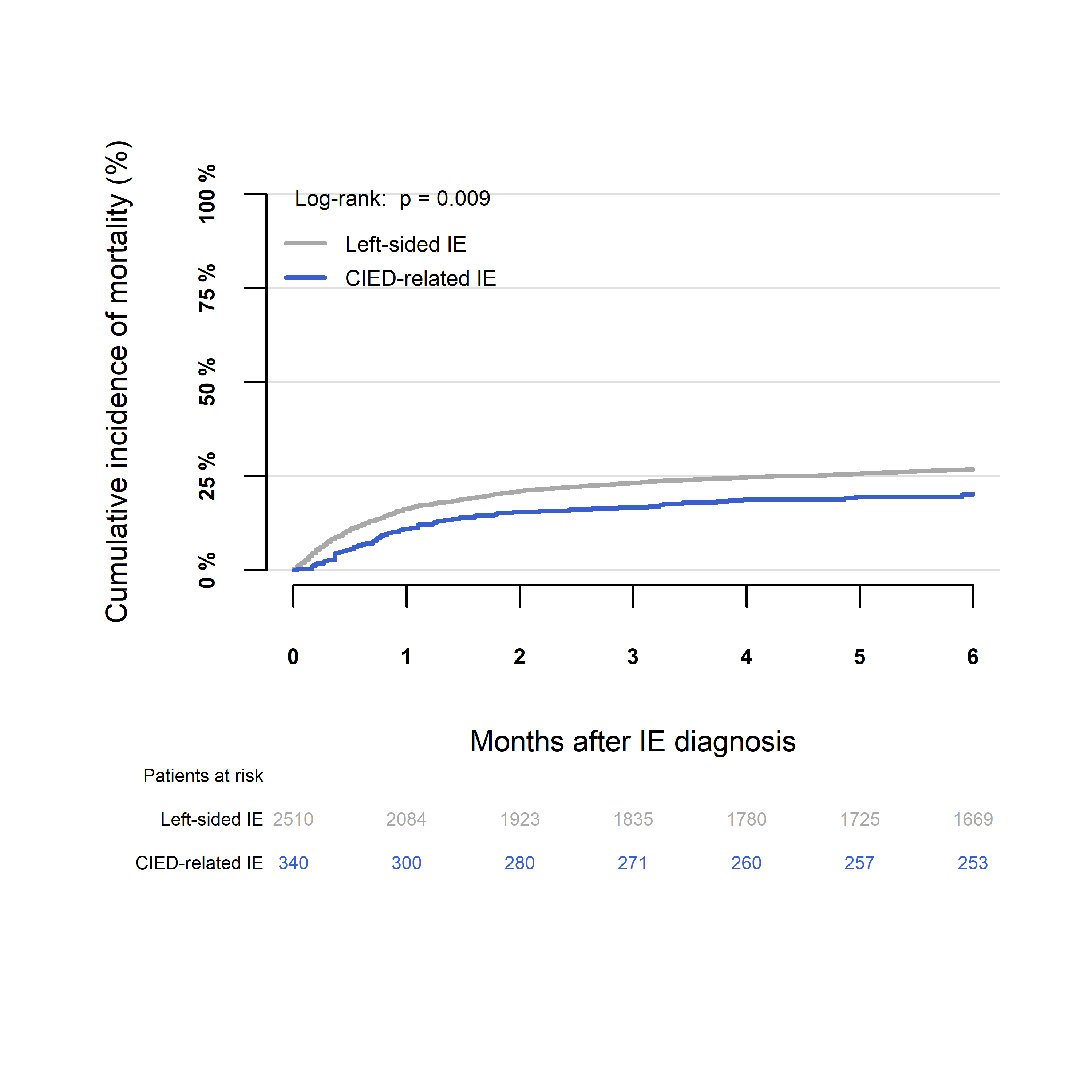


Abbreviations: CIED, cardiac implantable electronic device; IE, infective endocarditis

**Figure S2. Six-month cumulative incidence of relapse of bacteraemia, relapse of IE, reinfection of IE, the composite outcome, and all-cause mortality in patients with CIED-related and left-sided infective endocarditis with a CIED**


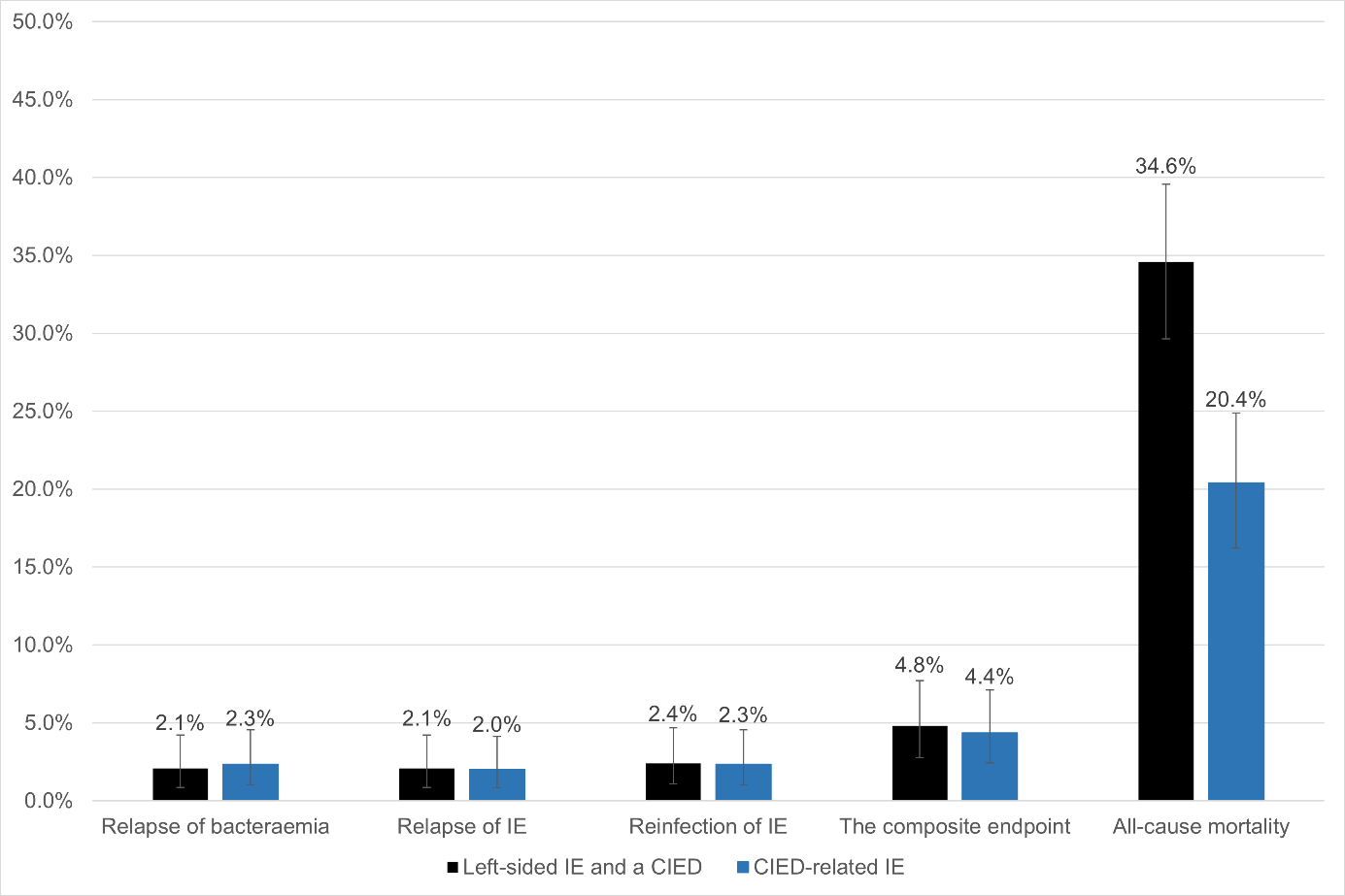


Relapse of bacteraemia and relapse of IE were defined as infection with the same microorganism, whereas reinfection of IE was defined as infection with a different microorganism. The composite outcome included all three, with death as a competing risk.

Abbreviations: CIED, cardiac implantable electronic device; IE, infective endocarditis

**Figure S3: Six-month cumulative incidence of all-cause mortality according to early CIED removal (≤7 days) and no CIED removal by day 7**


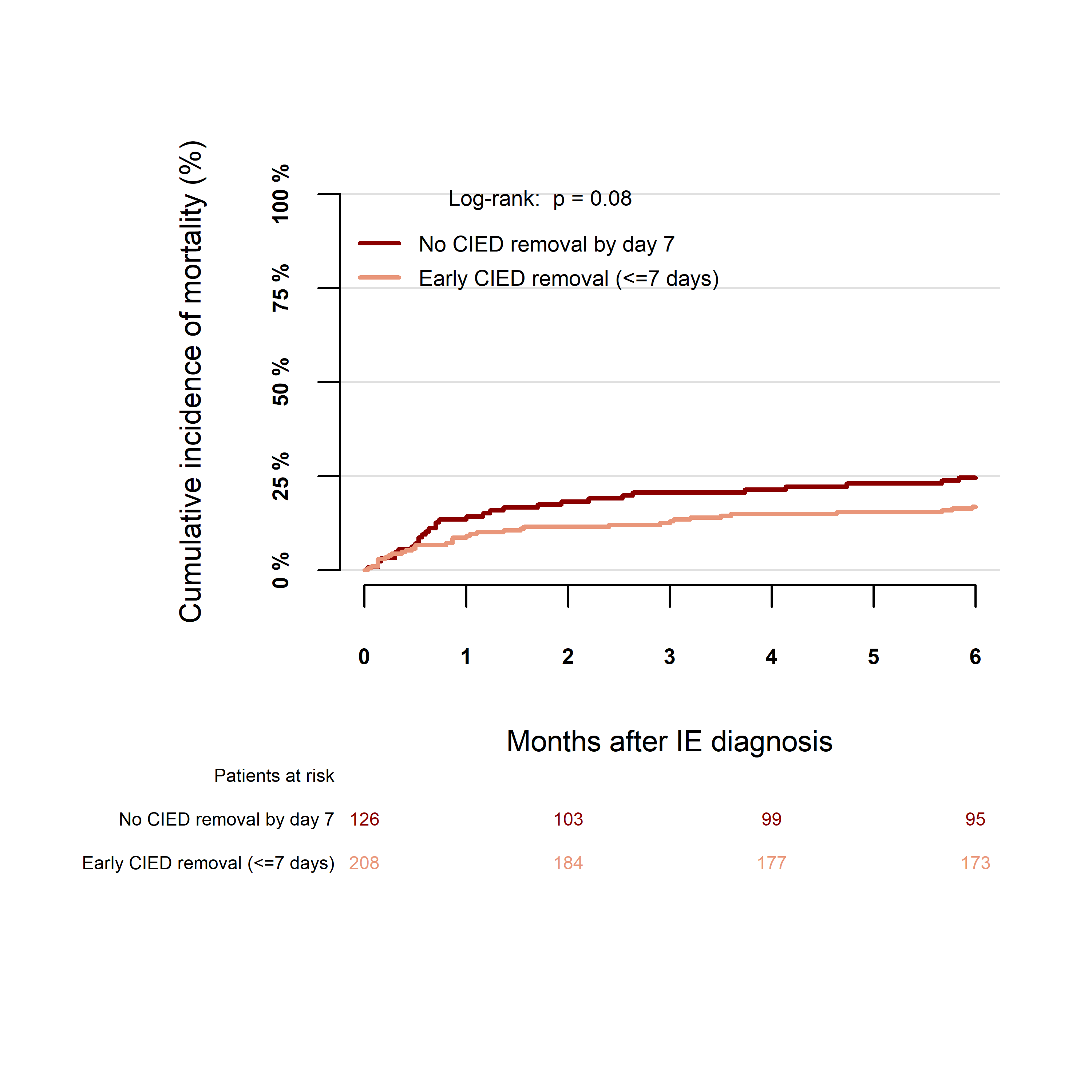


Abbreviations: CIED, cardiac implantable electronic device; IE, infective endocarditis
